# Older Transgender People’s Discrimination in Healthcare: A Scoping Review

**DOI:** 10.3390/geriatrics10060140

**Published:** 2025-10-28

**Authors:** Costas S. Constantinou, Monika Nikitara

**Affiliations:** 1 Department of Basic and Clinical Sciences, Medical School, University of Nicosia, Nicosia 1700, Cyprus; 2 Department of Life and Health Sciences, School of Sciences and Engineering, University of Nicosia, Nicosia 1700, Cyprus

**Keywords:** transgender older people, healthcare discrimination, scoping review

## Abstract

**Background**: Given the healthcare challenges older people and people who identify as LGBTQ+ are faced with, it becomes critical to explore how older transgender individuals experience discrimination in healthcare settings. **Objectives**: We followed the PRISMA guidelines for scoping reviews to map existing literature, and identify key themes, specific to transgender adults aged 65 and older and how they experience discrimination in healthcare. Eligibility criteria: This scoping review explored studies that focused on discrimination against transgender people older than 65 in any healthcare settings, which were published in English in the last twenty years. Sources of evidence: Evidence was extracted from Scopus, ProQuest Central, Health & Medical Collection, PubMed, CINAHL, Medline, and Psychology and Social Sciences databases. Charting methods: We used streams of search with specific keywords. Two researchers were involved in the screening of articles, coding and analysis. **Results**: The search showed that research focusing on transgender people older than 65 and discrimination in healthcare was rather limited. The findings revealed that older transgender individuals frequently anticipated or experienced discrimination in healthcare settings, resulting in service avoidance and adverse health outcomes. Despite sometimes reporting lower overt discrimination than younger cohorts, older trans people faced comparable levels of victimization, compounded by age-related vulnerabilities and socioeconomic marginalization. Structural barriers, such as misaligned documentation, lack of provider competence, and financial constraints, further hindered access to healthcare. However, the presence of empathetic, culturally competent providers and access to LGBTQ+-specialized services might improve care engagement and outcomes. **Conclusions**: This review concluded that targeted policy reforms, inclusive clinical practices, and community-based support systems were essential to address these disparities. It called for greater institutional accountability and interdisciplinary research to ensure safe, affirming, and equitable healthcare for aging transgender populations.

## 1. Introduction

Gender and sexual minorities constitute an important and growing portion of the global population. The acronym LGBTQ+—which stands for Lesbian, Gay, Bisexual, Transgender, Queer or Questioning, and others—represents a diversity of identities and orientations. Therefore, understanding the needs and challenges of this community is essential, particularly within healthcare systems that often do not accommodate or acknowledge these differences adequately.

There is evidence to suggest that people who identify as LGBTQ+ frequently encounter discrimination, stigma, and systemic barriers in healthcare settings [1,2,3]. Specifically, studies have documented that LGBTQ+ individuals experience a range of challenges such as fear of disclosing their identity, breach of confidentiality, direct and indirect discrimination, medical misconceptions, and healthcare provider bias. A notable investigation by Bachmann and Gooch [4] revealed the extent of these issues, with LGBTQ+ people reporting various negative experiences: one in eight had experienced some form of discrimination in healthcare; one in four had been subjected to inappropriate curiosity or questioning from medical staff; and transgender individuals were particularly vulnerable, with one in three reporting unequal treatment and nearly half experiencing intrusive questioning about their gender identity.

Furthermore, LGBTQ+ individuals—especially transgender people and LGBTQ+ people of colour—report more frequent and more severe instances of discrimination [5,6]. These challenges are not only psychological or emotional but have concrete effects on health outcomes. For instance, LGBTQ+ populations face disparities across several areas of health, including mental health, sexual and reproductive health, and cancer care [7]. These disparities often result from both individual-level bias and structural inequalities embedded in healthcare institutions.

The root causes of these healthcare disparities are multifaceted. One major contributor is the attitudes and behaviours of healthcare professionals. Research has shown that some practitioners still view sexual orientation or gender identity as irrelevant to a patient’s overall health or, worse, believe that these identities can or should change [8]. Additionally, many clinicians feel inadequately equipped or uncomfortable discussing gender or sexual identity with patients, which can hinder effective communication and lead to suboptimal care [9]. Interestingly, even physicians who identify themselves as LGBT have experienced discrimination, harassment and social isolation by other colleagues [10]. The authors also highlight that few had any formal education and that LGBT issues should be learned and discussed in medical schools. Compounding this issue is the fact that LGBTQ+ topics remain underrepresented in most medical and nursing curricula, leaving healthcare providers ill-prepared to address the specific needs of LGBTQ+ patients [11].

Importantly, LGBTQ+ healthcare professionals themselves often conceal their identities in the workplace due to fear of discrimination, further perpetuating a culture of silence and exclusion [12]. These systemic issues create an environment in which LGBTQ+ individuals are either discouraged from seeking healthcare or are left vulnerable when they do.

It is important to emphasize that LGBTQ+ and trans people are not passive recipients of discrimination, trapped in endless suffering without any tools to adapt. Instead, there is evidence to suggest that they have developed their own mechanisms of coping with such challenges. For example, as Ben-Lulu [13] showed, liturgies and prayers aiming to address LGBTQ+ concerns for medical treatments were turned into tools of resilience. These practices affirmed LGBTQ+ identities while positioning them as social agents who reshape spiritual life to meet their needs. Similarly, Blus-Kadosh and Hartal [14] demonstrated how patient activism drove trans-inclusive primary care. By asserting their unique experiential knowledge, trans patients worked with doctors in partnership to co-create more equitable health systems, ensuring that care was not only accessible but affirming. For instance, doctors used trans patients as a source of information rather than making assumptions about their body and needs. These practices highlighted the agency of trans communities in confronting systemic discrimination. At the same time, LGBTQ+ identities should not be understood in isolation. Instead, they are shaped by religion, ethnicity, class, and other intersecting factors that inform both marginalization and resilience [15,16]. Recognizing these intersections is crucial for understanding how LGBTQ+ lives are lived, negotiated, and transformed across diverse cultural and social contexts [17].

From this overview, it is clear that LGBTQ+ individuals are at risk of experiencing discrimination in healthcare environments, with transgender individuals facing particularly pronounced challenges, although they are, at the same time, agents adjusting to challenging situations. The next critical question is whether such discrimination becomes more acute for LGBTQ+ individuals aged 65 and older.

Aging introduces a new layer of vulnerability, as older adults already face numerous barriers in accessing healthcare. Ageism within healthcare has been documented, with older patients often experiencing negative attitudes and stereotypes [18]. When ageism intersects with LGBTQ+ discrimination, the result is compounded marginalization. Older LGBTQ+ adults may face unique stressors, including isolation, lack of social support, and increased likelihood of chronic illness [19]. These individuals are also more likely to rely on formal healthcare services due to estrangement from families or absence of children.

Research focusing specifically on older transgender individuals reveals an even grimmer picture. A qualitative study by Rosati, Pistella and Baiocco [20] found that older trans adults often delay or forgo medical care out of fear of mistreatment. Many reported being misgendered, denied access to appropriate medical interventions, or subjected to curiosity that borders on voyeurism. Another study by Fredriksen-Goldsen et al. [21] noted that older transgender people face high levels of psychological distress, largely attributable to cumulative lifetime experiences of discrimination and inadequate access to gender-affirming healthcare.

A particular concern is long-term care facilities, where older LGBTQ+ individuals may be forced back into the closet due to homophobic or transphobic staff and residents [22]. Some report having to conceal their identity to avoid verbal abuse or denial of care. Moreover, very few residential care facilities have LGBTQ+ affirming policies, and staff training on these issues remains minimal.

Given the intersection of age and gender identity, it becomes critical to explore how older transgender individuals specifically experience discrimination in healthcare settings. Such an exploration not only helps illuminate the complex web of factors that influence health outcomes but also points the way toward more inclusive policies and practices.

## 2. Methods

### 2.1. Justification for a Scoping Review

This study could potentially be either a systematic review or a scoping review. To determine the most appropriate approach, we referred to the guidelines by Munn et al. [23]. Munn et al. outlined the purposes and indications for both review types. According to their framework, systematic reviews are primarily conducted to synthesize international evidence related to specific research questions, with the goal of informing practice, policy, and future research. In contrast, scoping reviews are preferred for mapping emerging or underexplored areas of evidence—particularly when the research question is still broad or when the available literature is too limited or heterogeneous to support a systematic review.

With this distinction in mind, we conducted a preliminary literature search to assess the scope of existing research. We found that studies examining discrimination against transgender individuals over the age of 65 in healthcare settings were limited. Based on this finding, we concluded that a scoping review was more suitable.

After completing the review and identifying all studies relevant to our objectives, we revisited our initial decision. Before beginning the coding and analysis phase, we reflected again on the review type and reaffirmed that a scoping review was indeed the most appropriate approach.

### 2.2. Research Objectives

Although research questions are typically not used for scoping reviews, we relied on the PICO [24] guidelines for qualitative studies. That is, P = Population/Problem, I = Phenomenon of Interest, Co = Context. The ‘Population’ that this review focuses on are people who identify as transgender and are older than 65. ‘Trans is a general term for people whose gender is different from the gender assigned to them at birth’ [25].

The ‘Phenomenon of interest’ is experiencing any form of discrimination and the ‘Context’ is healthcare. Based on PICo guidelines, we have formulated the following research objectives: (a) What is the evidence on discrimination against transgender people who are older than 65 in healthcare, (b) How do older trans experience discrimination in healthcare.

### 2.3. Protocol

We have followed the PRISMA extension for scoping reviews (PRISM-ScR) checklist as published by Tricco et al. [26]. This checklist has 27 items in total including some which are only application to systematic reviews and a couple of optional ones. In some instances, we decided to merge items that would be a repetition if addressed them separately. For example, ‘characteristics and results of sources of evidence’ are two separate items in Tricco et al.’s checklist. Also, we have included critical appraisal tables, although this item is optional.

### 2.4. Eligibility Criteria

The studies in this review were selected had to meet specific inclusion criteria reflecting the PICo guidelines, as described in the above, and as per the Table 1.

### 2.5. Information Sources

We used those databases which provided breadth of resources, were most relevant to our research question and our institution had access to. Given this, we searched the databases Scopus, ProQuest Central, Health & Medical Collection, PubMed, CINAHL, Medline, and Psychology and Social Sciences databases. Google Scholar was used as a supplementary resource and consulted after searching of the databases above was completed [27].

### 2.6. Search

To search for any studies that met our objectives, we used the keywords and the limits as per the Table 2.

### 2.7. Selection of Sources of Evidence and Data Charting Process

To ensure quality of screening and review, once the articles were identified, two authors reviewers screened the articles independently and then met to discuss their results. Having finalised the list of the articles that were included in the review for full evaluation, the two reviewers did the coding and a preliminary synthesis of results before finalizing the analysis.

### 2.8. Data Items

We abstracted data on article characteristics (date and language of publication, type of research and methods, country, sample size), study population (transgender people, 65 or older), settings (any healthcare settings), and discrimination (any form of discrimination relevant to access, treatment, information giving, communication).

### 2.9. Synthesis of Results

We summarized the types of settings, populations and sampling, types of research designs, and results for each of the article as per the Table 3. If we found a review, we checked if it used any empirical studies that we had not identified and looked for these studies in the databases. We then read results multiple times, along the lines of the ‘Framework Method’ as outlined by Ritchie and Spencer [28], and coded and constructed themes.

### 2.10. Critical Appraisal of Individual Sources of Evidence

For the critical appraisal of the studies selected for detailed review and coding, we relied on the Joanna Briggs Institute’s critical appraisal tools [29], which were relevant to the type of studies included (Table 4 and Table 5), with the exception of one article that was closer to a narrative review, for which we used the SANRA scale [30] (Table 6).

### 2.11. Reflexivity

Although reflexivity is not listed in the PRISMA guidelines for scoping reviews, we agreed with the suggestion by one of our reviewers that, as authors of this paper who conceived the aims and were involved in all steps, it is important to reflect on our background, considering how it might have impacted the selection of studies, the coding process, the findings, and our conclusions. We have discussed rigorously between ourselves, and we have concluded that we had the privilege of falling between two stools. On the hand, neither of us identify with the LGBTQ+ community in the sense that our gender identity does not fall in the spectrum of the community’s identities. This helped us being rather distanced from implicitly selecting papers that showed discrimination against older trans people. However, one could argue that this could have implicitly caused us to focus more on studies that did not find serious incidents of discrimination. On the other hand, we do identify ourselves as allies of the LGBTQ+ community, standing for their rights and freedoms to self-identify and express their gender, and we promote societies and healthcare settings that are inclusive, welcoming, affirming, and psychologically safe. On this note, we are confident that we have been as objective as possible during our selection process, coding and analysis, which we conducted independently from each other, as we explained in our methodology, to tackle any possible biases.

### 2.12. Registration

This scoping review is registered to OSF (Open Science Framework) with number 10.17605/OSF.IO/P9VYE.

## 3. Results

### 3.1. Selection of Sources of Evidence

**Figure 1:**
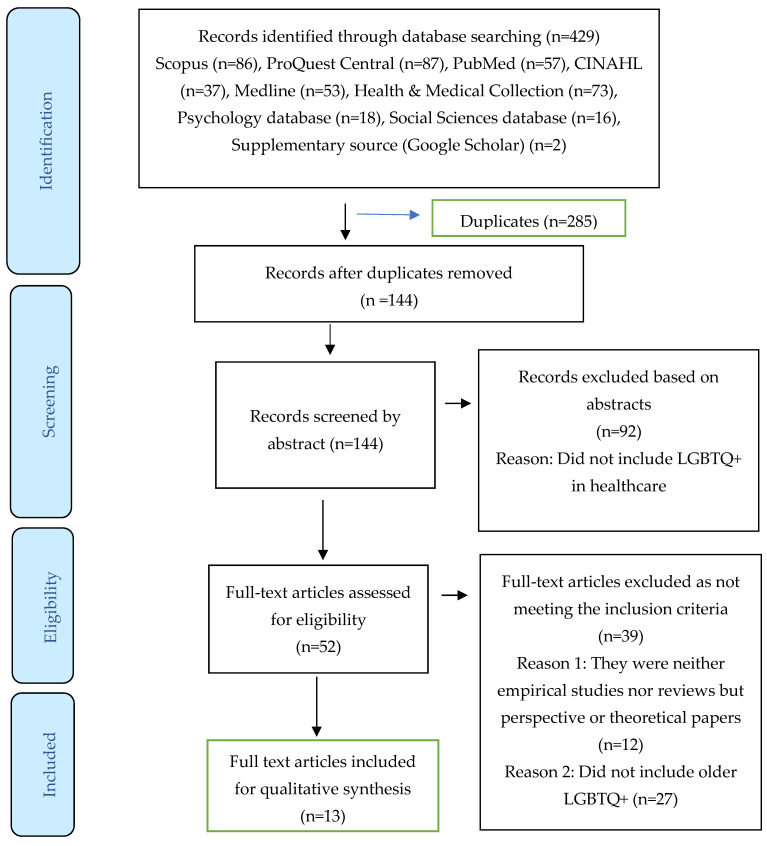
PRISMA Scoping Review flowchart of the process of identification, screening, eligibility and inclusion [26].

Searching for evidence as our search strategy, we initially found 429 resources (Figure 1). After duplicates were removed, a total of 144 were identified and screened against our inclusion and exclusion criteria. Reviewing the abstracts at this stage, we excluded 92 resources because they were not including people identifying with the LGBTQ+ community. The remaining 52 resources were fully reviewed, and we decided to exclude 12 resources because they were neither empirical studies nor reviews, and 27 because they explicitly studied people younger ages. Therefore, 13 articles were included in this scoping review study. These 13 articles are summarized in Table 3, and critically appraised in Table 4, Table 5 and Table 6.

**Characteristics and results of sources of evidence**

**Table 3:** Results of individual sources of evidence.

First Author, Date, Reference	Country/ National or Regional	Date of Collection, Survey/Recruitment	Analysis, Analyzed *n*, Sample Age	Data Collection Method	Summary of Results Relevant to This Scoping Review
Adan, 2021 [31]	USA, New York	Date not specified	Qualitative, 19 participants, 65 or older	Semi-structured interviews	Authors discussed the following: fear of mistreatment, isolation and loneliness, increased vulnerability to financial stressors, perceived lack of agency, healthcare system and provider inclusivity. The findings of the study revealed that older trans people were afraid of mistreatment, especially regarding the lack of affirmation of their gender identity. Older trans people mentioned examples of microaggressions such as wrong pronouns or even more serious incidents like physical and sexual abuse.
Wirth, 2020 [32]	N/A	N/A	Review	N/A	Older trans people are largely invisible and are less likely to seek special medical and nursing help than cisgender people. Interestingly, in acute conditions or in paternalistic settings their gender might be reassigned because of hormones’ interactions. As a result, older trans people very often experience mental burden with increased rates of depression and anxiety. Other concerns include lack of control over their care, use of language in medical settings, medical records not accommodating gender identity etc. These experiences of discrimination in healthcare were exacerbated by problematic experiences in other settings. Specifically, lack of social interaction, estrangement from partners and other family members, financial insecurity due to transphobia, lack of control over one’s treatment at end of life or in nursing homes, challenge for self-acceptance of transgender identity.
Lampe, 2025 [33]	USA	2021–2022	Qualitative, 47 participants, 65 or older	Semi-structured interviews	Two of the themes related to trans and healthcare. First, engaging with behavioural healthcare practitioners who offer compassionate, patient-centre care. older trans people were very happy when their carers were very knowledgeable of transgender and gender non-conforming-older-adults community terminology and had work with trans patients before. Also, these carers were understanding and showed empathy. This was also reflected in carers’ ability to listen without interruptions. Second, receiving equitable access to gender-affirming care and social services. When carers were understanding and knowledgeable they provided all necessary support to facilitate the healthcare procedures, including time management and ways to overcome the legal and medical barriers during their gender transition.
Kattari, 2016 [34]	USA	Secondary data from the 2010 National Transgender Discrimination Survey	Quantitative, 5885 participants, Ages below 35, 35–49, 50–64, 65 or above 114 participants older than 65	Survey	All participants from all ages indicated that they experienced discrimination, harassment and victimization in healthcare. Interestingly, in general participants older than 65 were less likely than any of the other age cohorts to experience discrimination and harassment. As an indication, 6.1% of participants older than 65 experienced discrimination as compared to 27% of the participants younger than 35. However, they experienced victimization as much as the younger cohorts. The authors explained the fact that older participants experienced less discrimination was possibly due to and under reporting and to generational differences in how participants defined and discussed discrimination.
Bloemen, 2019 [35]	USA, New York	2016	Qualitative, 26 participants	Focus Groups	The study did not focus exclusively on trans and healthcare but it encompassed lesbian, gay, bisexual and forms of neglect and abuse in various settings. Regarding the results relating to this review, the main experiences of discrimination or perceived discrimination had to do with not seeking help due to the fear of not being accepted and respected by the healthcare providers. Others raised the issue of problematic communication with healthcare providers who do not pay the necessary attention, and to being double victimized because of ageing and their gender identity. Other issues related to ageing were: reduced access to housing, avoidance of long-term care, increased vulnerability due to physical and cognitive impairment.
Koller, 2023 [36]	USA	Date not specified	Qualitative, 20 participants, 50 or older including older than 65	Interviews	The study was about the general experience of transgender people. Among other things, participants expressed concerns with healthcare, which were interwoven with other experiences such as the process of accepting themselves, coming out, passing as transgender etc. The factors that helped were support within the transgender community, and by closed others.
Banerjee, 2021 [37]	India	Date not specified	Qualitative, 10 participants, 60 or older	Interviews	Participants experienced many challenges during the COVID-19 pandemic. Specifically, they experienced marginalization, survival threats, and double burden due to age and gender identity. Participants clarified that they considered as ‘second priority’ for health and legal services, including access to medications, medical protective equipment, and testing. Participants experienced social and health stigma strongly during the COVID-19 pandemic.
Dickson, 2022 [38]	USA	2018	Quantitative, 789 participants, 178 participants were 63 or older	Online survey	The study focused on the utilization of long-term care services. Those identified as queer/trans reported more discrimination than gay men. 78.6% anticipated discrimination, with verbal harassment being the most common form. Trans along with those identified as queer, questioning, nonbinary, were more likely to anticipate more discrimination. Overall, there was a negative correlation between discrimination and willingness to utilize services in the future (i.e., higher discrimination, less willing to utilize), while 76.3% participants clarified that they would utilize the services in the future if they were offered by LGBTQ+ providers. The authors did not present or discuss any findings regarding age and discrimination.
Kattari, 2021 [39]	USA	2015 United States Transgender Survey	Quantitative, 27,715 participants	Secondary data analysis	The study explored the experiences of transgender and gender diverse adults with accessing a trans knowledgeable primary care physician. In general, participants’ visit to a physician dependent on their background. Specifically, white individuals, those who were more educated, those with more disabilities and older were more likely to visit a physician. Also, older age was associated with more chances to have a transgender knowledgeable physician. The authors concluded that the link between older and having a physician or a transgender knowledgeable physician has to do with the fact that people as they age have access to more resources and they receive more message about the importance of regular visits. The authors did not specify the age cohorts they used.
Klein, 2024 [40]	USA	2015 United States Transgender Survey	Quantitative, 27,715 participants, 3724 participants were 50 or older	Secondary data analysis	The study investigated the factors contributing towards contemplating suicide among older trans people. The five factors were: workplace issues, interactions with professionals, using public services, safety issues, and socioeconomic disadvantages. About half of the participants (47.9%) reported problems they had with professionals, while 27.6% reported unequal treatment by a healthcare professionals due to being transgender. The authors did not split the participants in more age cohorts. About 12% said that they would avoid using publically-available services to minimize the risk of being harassed or victimized.
Kachen, 2020 [41]	USA	2015 United States Transgender Survey	Quantitative, 27,715 participants, 12.5% of the participants were 65 or older	Secondary data analysis	The general finding for all ages was that transfeminine and transmasculine were more likely to postpone utilization of healthcare services due to fear of being discriminated against more than non-binary people. The reason why non-binary participants delayed seeking help had to do with cost. Transfeminine participants reported highest rates of discrimination in healthcare (31%). The authors did not compare age cohorts.
Savage, 2023 [42]	USA	2017 national survey of LGBT Americans aged 45 or older AARP	Quantitative, 1762 participants, 264 were ‘gender expansive’ including trans, non-binary, gender fluid, intersex. The age cohorts 65–74 and 74+ were included in the study	Secondary data analysis	The study aimed to explore what are participants’ concerns about the potential for neglect, harassment, denial of services and identity disclosure in long-term care. Gender expansive people were more likely to be concerned about abuse, verbal or physical harassment, and forced to hide their identity as comparted with cisgender males. This was exacerbated with age because of the potential for needing more long-term care. Regarding gender identity, those who were concerned about social support were more likely to be concerned about abuse, verbal or physical harassment, and limited access to services. It seems that good social and family support were protective factors.
Rosati, 2021 [20]	Italy	2018–2019	Qualitative, 23 participants, aged 60 or older, the study did not split participants in gender identity categories.	Interviews	Many participants avoided seeking help due to the fear of being discriminated against. These expectations resulted from direct past experiences or from homophobic attitudes by healthcare professionals. Some participants experienced direct discrimination, especially those who were seropositive and were denied treatment. Participants expressed interest in visiting sexual-minority clinics.

**Critical appraisal within sources of evidence**

**Table 4:** JBI Critical Appraisal Checklist for Analytical Cross-Sectional Studies.

First Author, Date, Reference	Q1	Q2	Q3	Q4	Q5	Q6	Q7	Q8
Kattari, 2016 [34]	√	√	√	√	√	√	√	√
Dickson, 2022 [38]	√	√	√	√	√	√	√	√
Kattari, 2021 [39]	√	√	√	√	√	√	√	√
Klein, 2024 [40]	√	√	√	√	√	√	√	√
Kachen, 2020 [41]	√	√	√	√	√	√	√	√
Savage, 2023 [42]	√	√	√	√	√	√	√	√

**Table 5:** JBI Critical Appraisal Checklist for Qualitative Studies.

First Author, Date, Reference	Q1	Q2	Q3	Q4	Q5	Q6	Q7	Q8	Q9	Q10
Adan, 2021 [31]	√	√	√	√	√	No	Unclear	√	√	√
Lampe, 2025 [33]	√	√	√	√	√	No	Unclear	√	√	√
Bloemen, 2019 [35]	√	√	√	√	√	No	Unclear	√	√	√
Koller, 2023 [36]	√	√	√	√	√	No	Unclear	√	√	√
Banerjee, 2021 [37]	√	√	√	√	√	No	Unclear	√	√	√
Rosati, 2021 [20]	√	√	√	√	√	No	Unclear	√	√	√

**Table 6:** SANRA scale for Narrative Reviews.

First Author, Date, Reference	Q1	Q2	Q3	Q4	Q5	Q6
Wirth, 2020 [32]	√	√	No	√	√	√

### 3.2. Synthesis of Results

This scoping review systematically explores the lived experiences of older transgender individuals in relation to healthcare and adjacent institutional systems, drawing upon a diverse array of empirical studies. The collective findings elucidate a multifaceted and often adverse landscape characterized by fear, discrimination, resilience, and a marked imperative for inclusive, affirming care. The results are thematically organized below.

### 3.3. Anticipated Mistreatment and Experiences of Discrimination Within Healthcare Settings

Older transgender adults frequently encounter healthcare environments that are perceived as hostile, unwelcoming, or indifferent to their identities. The anticipation of discriminatory treatment—rooted in prior encounters with explicit denial of care or exposure to microaggressions—often discourages healthcare utilization [31]. These apprehensions are substantiated by numerous accounts across studies, revealing both overt mistreatment and more insidious forms of marginalization, ultimately contributing to avoidance of essential medical care and deterioration in physical and mental health outcomes [31,32].

Interestingly, data reveal that individuals aged 65 and older report lower rates of discrimination (6.1%) compared to those under 35 (27%) [34]. Kattari and Hasche [34] hypothesize that this discrepancy may reflect generational differences in the recognition, internalization, or articulation of discriminatory experiences, or potential underreporting among older cohorts. Nonetheless, levels of victimization remain comparable across age groups.

### 3.4. Social Isolation, Loneliness, and Mental Health Vulnerabilities

Older transgender individuals are disproportionately affected by social isolation, frequently resulting from familial estrangement or the dissolution of prior relationships following gender transition. This social disconnection is closely associated with elevated rates of depression, anxiety, and existential hopelessness [35].

These mental health challenges were exacerbated during the COVID-19 pandemic, wherein transgender individuals reported systemic de-prioritization within both healthcare and legal frameworks [37]. Limited access to personal protective equipment, medications, and diagnostic testing, coupled with heightened stigmatization, underscored their compounded vulnerability at the nexus of age and gender identity [37,42].

### 3.5. Structural Barriers to Gender-Affirming and Equitable Care

Significant structural impediments hinder older transgender individuals’ access to healthcare aligned with their gender identity. These barriers include the absence of inclusive or affirming language during clinical encounters, and a pervasive lack of provider competence regarding trans-specific healthcare needs [33].

Additional systemic challenges—such as prohibitive costs and extended wait times—further compound these disparities. Transfeminine individuals reported higher incidences of healthcare avoidance due to anticipated discrimination, whereas non-binary individuals more frequently cited financial constraints [41]. Conversely, care delivered through LGBTQ+-affirming organizations substantially increased willingness to engage with health services; in one study, 76.3% of respondents expressed readiness to utilize long-term care if it were provided by LGBTQ+-friendly institutions [38].

### 3.6. The Role of Affirming Care and Empathic Providers

Two interrelated factors emerged as critical to improving healthcare experiences: access to gender-affirming services and interaction with empathetic, culturally competent providers. Participants highlighted the importance of clinicians who possessed both experiential familiarity with transgender patients and a deep understanding of appropriate terminology and identity nuances [33].

Providers who demonstrated compassion, engaged in active listening, and fostered non-judgmental environments were perceived as facilitating safer, more trusting clinical interactions [33,38]. Moreover, these affirming practitioners often assumed advocacy roles—assisting with legal processes, expediting gender-affirming procedures, and dismantling institutional obstacles—thus significantly enhancing healthcare outcomes and patient engagement.

### 3.7. Discrimination as a Determinant of Service Utilization

A robust inverse correlation was observed between experiences of discrimination and willingness to utilize healthcare services. Anticipated discrimination, particularly within long-term care contexts, served as a powerful deterrent [20,31,34]. Among LGBTQ+ individuals, 78.6% expected some form of mistreatment—primarily verbal harassment—if they sought such services [38].

This apprehension was most acute among individuals identifying as queer, non-binary, or gender expansive. Many expressed fear of neglect, identity disclosure, or exposure to harassment in institutional settings [35,42]. Notably, these fears intensified with age, as the need for long-term care became more imminent [32,42].

### 3.8. Compounded Vulnerabilities and Socioeconomic Marginalization

The intersection of gender identity with other axes of vulnerability—including socioeconomic precarity, age-related decline, and systemic exclusion—exacerbated risks for older transgender individuals. Respondents detailed adverse experiences with public institutions, employment discrimination, and unsafe living environments, all of which were linked to suicidal ideation and psychological distress [40].

Nearly half (47.9%) reported negative interactions with professionals, and over one-quarter (27.6%) experienced discriminatory treatment by healthcare providers [40]. Approximately 12% avoided public services entirely, citing concerns about harassment or discrimination. Additionally, those with age-related cognitive or physical impairments faced heightened barriers, diminishing their capacity for self-advocacy and compounding their marginalization [40].

### 3.9. Protective Factors and Community-Based Resilience

Despite pervasive challenges, several protective factors were identified. Strong ties to LGBTQ+ communities and supportive social networks were associated with improved mental health outcomes and greater ease navigating healthcare systems [36]. Older transgender individuals were also more likely to have established relationships with trans-affirming healthcare providers, reflecting both accumulated resources and a greater prioritization of ongoing care [33,39].

Participants consistently emphasized the value of LGBTQ+-specific clinics, perceiving them as safer and more respectful environments—particularly vital for individuals living with stigmatized conditions such as HIV [20,36,38].

### 3.10. Summary

This review reveals an enduring pattern of healthcare inequities experienced by older transgender adults, shaped by a complex interplay of discrimination, fear, social marginalization, and systemic exclusion. Nonetheless, the data also underscore the transformative potential of affirming care environments and compassionate, knowledgeable providers. To promote health equity and uphold the dignity of aging transgender populations, it is imperative to address structural barriers and expand access to inclusive, culturally competent, and gender-affirming services.

## 4. Discussion

The findings of this scoping review confirm, extend, and in some respects complicate existing understandings of healthcare discrimination experienced by LGBTQ+ populations, particularly transgender individuals. A critical comparison of the literature reviewed and the empirical data reveals important continuities, emergent divergences, and underexplored nuances in the lived experiences of older transgender adults.

Consistent with existing research [1,2], the findings reaffirm that transgender individuals encounter systemic discrimination within healthcare environments. This aligns with earlier studies documenting experiences of stigma, mistreatment, and exclusion in clinical settings [3,4]. For instance, the reviewed literature emphasizes the prevalence of microaggressions, inappropriate curiosity, and denial of care, particularly for transgender individuals [4,20]. The current review similarly found that many older transgender individuals anticipate or recall discriminatory treatment, often resulting in healthcare avoidance—a pattern also well-documented in younger LGBTQ+ cohorts.

However, a notable divergence emerges in reported rates of discrimination across age groups. The findings indicate that individuals aged 65 and older sometimes report lower rates of discrimination (6.1%) compared to their younger counterparts under 35 (27%). This finding appears counterintuitive given the literature’s strong emphasis on the compounded marginalization of older transgender adults [19,20]. One possible explanation is the generational difference in the perception or articulation of discrimination, as older individuals may internalize or normalize past mistreatment due to lifetime habituation to systemic bias [16]. Alternatively, underreporting could be a function of resilience mechanisms or selective healthcare engagement with only trans-affirming providers. These hypotheses suggest a complex interplay between subjective reporting and structural realities that require more granular investigation.

The review further substantiates the literature’s emphasis on social isolation as a significant stressor for older transgender individuals [19]. As noted by previous scholars, the intersection of age, gender identity, and relational estrangement increases the risk of loneliness and poor mental health outcomes [22]. The findings reinforce these concerns, documenting elevated rates of depression, anxiety, and existential distress among older trans adults—challenges that were exacerbated during the COVID-19 pandemic. This aligns with earlier observations about the de-prioritization of LGBTQ+ needs during health crises [20], but the findings here offer a more acute sense of pandemic-era marginalization, illustrating how overlapping vulnerabilities—such as age, identity, and public health neglect—create unique layers of risk.

Moreover, the current findings elaborate on the structural and procedural barriers that hinder equitable access to gender-affirming care. While previous literature points to a lack of clinical training and institutional support [8,11], the review deepens this critique by highlighting practical challenges such as mismatched documentation, high costs, and long wait times. These barriers are particularly pronounced for transfeminine and non-binary individuals, underscoring the inadequacy of “one-size-fits-all” approaches in trans healthcare. This suggests that systemic reform must not only address attitudinal bias but also the operational mechanisms that perpetuate exclusion.

One critical advancement this study contributes is its attention to the differentiated experiences within the transgender umbrella. While earlier works often treat trans individuals as a homogenous category [5,6], this review disaggregates data by identity (e.g., transfeminine, non-binary), revealing important distinctions in healthcare avoidance, cost barriers, and vulnerability to discrimination. For example, non-binary individuals reported greater financial barriers to care, while transfeminine individuals were more likely to anticipate mistreatment—highlighting the necessity for intersectional, identity-specific policy responses. The intersectionality and its linked with gender identity has been identified by other scholars [15,16].

The role of affirming care and empathetic providers emerges as a major theme in both the literature and the findings, reinforcing the work of Sekoni et al. [9] and Eliason et al. [10], who emphasize provider attitudes and competence as determinants of care quality. This review amplifies these insights, showing how affirming clinicians not only deliver better care but also act as advocates—assisting with legal documentation, facilitating access to trans-specific services, and building trust in otherwise hostile systems. Such providers serve as critical counterweights to institutional exclusion, especially for older transgender patients who may have fewer social supports.

Another significant contribution of the review is its focus on long-term care—a setting that receives limited attention in earlier research but is increasingly relevant for aging LGBTQ+ populations. While Hash and Rogers [22] identified the risk of older LGBTQ+ individuals “re-entering the closet” in residential facilities, the findings here bring specificity and urgency to those concerns. The widespread fear of harassment, identity suppression, and neglect in these settings acts as a powerful deterrent to care-seeking. The data showing that 78.6% of respondents expect mistreatment in long-term care suggests a pervasive crisis of trust that cannot be addressed solely through provider education; systemic policy reform and institutional culture change are necessary.

The review also introduces socioeconomic precarity and institutional disenfranchisement as co-determinants of healthcare inequity—issues that are underexplored in much of the literature. While previous studies acknowledge that LGBTQ+ people face higher rates of poverty and housing insecurity [19], the current findings illustrate how these factors intersect with cognitive and physical decline to further marginalize older transgender adults. Importantly, 47.9% of participants reported negative interactions with professionals outside healthcare, and over a quarter (27.6%) experienced discrimination from healthcare providers, with some avoiding public services altogether. These data points underscore the broader institutional failures that extend beyond medical care into housing, employment, and social services, thus framing healthcare discrimination as part of a larger matrix of structural exclusion.

Despite the prevalence of these challenges, the review documents protective factors and community-based resilience—a relatively underemphasized dimension in the literature. While some studies, such as Sabin et al. [43], note that LGBTQ+ professionals conceal their identities, few have explored the compensatory strategies developed by older transgender individuals to navigate exclusion. The current findings show that strong connections to LGBTQ+ communities, as well as long-standing relationships with affirming providers, can buffer against institutional neglect and improve mental health outcomes. This suggests that community-based healthcare models—particularly those offering LGBTQ+-specific services—play a crucial role in mitigating the harms of systemic discrimination. Community-based protective factors have been documented in other studies, including patient advocacy and the role of religious practices in dealing with discrimination and exclusion [14,16].

As broader implications, this review has illustrated how micro-level experiences of older transgender adults—such as healthcare avoidance, social isolation, or mistrust of institutions—reflect broader structural inequalities shaping trans people’s lives. Age emerges as a critical category, intersecting with gender identity, socioeconomic background, and health status to produce layered vulnerabilities. At the same time, the aging body introduces new health needs, complicating access to affirming care and reshaping perceptions of identity, resilience, and vulnerability. These findings reveal how individual narratives embody larger systems of exclusion, resilience, and reform.

In sum, this scoping review not only reinforces key themes from the existing literature—such as healthcare discrimination, social isolation, and the need for provider education—but also extends current understanding by highlighting age-related disparities, identity-specific barriers, and the impact of institutional structures beyond healthcare settings. It contributes new insights into generational dynamics in discrimination reporting, the differentiated experiences of non-binary and transfeminine individuals, and the urgent need for inclusive long-term care models. These findings call for more targeted, intersectional research and robust policy interventions that center the voices and needs of older transgender adults within healthcare reform agendas, taking into consideration intersectionality and multiple vulnerabilities.

## 5. Limitations

This scoping review has some limitations. First, the number of studies included is relatively small, and most originate from one or two countries. Second, because the existing research focuses on specific countries, we lack a comprehensive understanding of how transgender people experience discrimination in healthcare worldwide. Third, the studies reviewed are primarily surveys, with only a few qualitative investigations. Incorporating data from mixed-methods research, observational studies, and randomized controlled trials would provide a more nuanced understanding of the issue, reducing reliance on self-reported information.

Despite these limitations, this scoping review offers valuable insights, underscoring that discrimination against transgender people in healthcare is a real and ongoing problem—one that can be addressed through well-trained healthcare professionals who are knowledgeable and experienced in LGBTQ+ issues.

## 6. Conclusions

This scoping review emphasizes the complex challenges older transgender adults encounter within healthcare. While broader research documents discrimination, stigma, and inadequate care among LGBTQ+ populations, this review highlights vulnerabilities unique to trans people and aging. These include heightened social isolation, limited access to affirming services, and compounded socioeconomic disadvantages. Although older transgender people may report lower levels of overt discrimination than younger cohorts, they still face considerable barriers, often stemming from structural shortcomings and insufficient provider knowledge. The review also identifies protective factors that can reduce these disparities, particularly access to gender-affirming healthcare, support from informed and compassionate providers, and strong community networks. Such elements play a vital role in building trust, improving health outcomes, and enhancing psychological well-being.

Findings further point to the need for systemic reforms, such as strengthening provider education on transgender health, implementing inclusive policies in long-term care, and addressing intersecting inequalities like poverty and disability. Future research should adopt intersectional approaches that reflect diverse identities and the evolving needs of aging populations. Ultimately, ensuring equitable and safe care for older transgender adults requires institutional accountability, inclusive policies, and sustained investment in LGBTQ+-specific healthcare—both a matter of health equity and moral responsibility.

## Figures and Tables

**Table 1:** Inclusion/exclusion criteria.

Inclusion	Studies that explored the experience or transgender people in any healthcare settings, including medicine, nursing, physiotherapy, psychology etc. Studies that focused on LGBTQ+ but included transgender people were also considered. Studies that focused or included transgender people 65 or older. Published in the last twenty years, namely 2004–2025 in English in any country.
Exclusion	Studies that did not include transgender people. Studies that explicitly and exclusively included participants younger than 65. Studies conducted for fields other than medicine, nursing, and other allied healthcare. Published before 2004 and/or in a language other than English.

**Table 2:** Streams of search with the use of keywords.

(“Older adults” OR “Elderly” OR “Seniors” OR “65 and older” OR “Aging population”) AND (“Transgender” OR “Non-binary” OR “Gender nonconforming” OR “LGBT” OR “Gender diverse”) AND (“Discrimination” OR “Bias” OR “Stigma” OR “Health disparities”) AND (“Healthcare” OR “Medical care” OR “Health services” OR “Doctor” OR “Nursing” OR “Hospital”) AND (“Experiences” OR “Perceptions” OR “Views”)

## Data Availability

No new data were created or analyzed in this study. Data sharing is not applicable to this article.
